# Supplementary material for: Who are the ‘social Darwinists’? On dispositional determinants of perceiving the social world as competitive jungle
Source: PLoS One. 2021 Aug 11;16(8):e0254434. doi: 10.1371/journal.pone.0254434 (PMC8357178; doi:10.1371/journal.pone.0254434)
Supplement: S1 Appendix — (DOCX) [file pone.0254434.s005.docx]

**S1 Appendix**

**Competitive Jungle Belief**

1. My knowledge and experience tells me that the social world we live in is basically a competitive ’jungle’ in which the fittest survive and succeed, in which power, wealth, and winning are everything, and might is right

2. It's a dog-eat-dog world where you have to be ruthless at times

3. Life is not governed by the ’survival of the fittest’, we should let compassion and moral laws be our guide (*reverse-scored).*

4. You know that most people are out to ’screw’ you, so you have to get them first when you get the chance

5. The best way to lead a group under one's supervision is to show them kindness, consideration, and treat them as fellow workers, not as inferiors (*reverse-scored)*

6. If one has power in a situation, one should use it however one has to in order to get one's way

7. All in all it is better to be humble and honest than important and dishonest (*reverse-scored)*

8. Money, wealth and luxury are what really count in life

9. Charity (i.e., giving somebody something for nothing) is admirable, *not* stupid (*reverse-scored)*

10. It is much more important in life to have integrity in your dealings with others than to have money and power (*reverse-scored)*

11. If it's necessary to be cold blooded and vengeful to reach one's goals, then one should do it

12. Honesty is the best policy in all cases (*reverse-scored).*

13. There is really no such thing as ’right’ and ’wrong’. It all boils down to what you can get away with.

14. Do unto to others as you would have them do unto you, and never do anything unfair to someone else (*reverse-scored)*

15. Basically people are objects to be quietly and coolly manipulated for one's own benefit
